# Structural and Luminescent Properties of WO_3_-Modified Eu^3+^-Doped Borosilicate Glasses

**DOI:** 10.3390/ma19214478

**Published:** 2026-10-24

**Authors:** Margarita Milanova, Aneliya Yordanova, Margit Fabian, Lyubomir Aleksandrov, Reni Iordanova, Petia Petrova, Maria Gancheva

**Affiliations:** 1 Institute of General and Inorganic Chemistry, Bulgarian Academy of Sciences, G. Bonchev Str., Bld. 11, 1113 Sofia, Bulgaria; margi@svr.igic.bas.bg (M.M.); lubomirivov@gmail.com (L.A.); reni@svr.igic.bas.bg (R.I.); m.gancheva@svr.igic.bas.bg (M.G.); 2 National Centre of Excellence Mechatronics and Clean Technologies, 8 Bul., Kl. Ohridski, 1756 Sofia, Bulgaria; 3 HUN-REN Centre for Energy Research, 29-33 Konkoly Thege Street, 1121 Budapest, Hungary; fabian.margit@ek.hun-ren.hu; 4 Institute of Optical Materials and Technologies “Acad. Jordan Malinowski”, Bulgarian Academy of Sciences, Blvd. Akad. G. Bonchev Str., Bld. 109, 1113 Sofia, Bulgaria; petia@iomt.bas.bg

**Keywords:** glass, rare earths, photoluminescence, density

## Abstract

In this study, the influences of WO_3_ on the structure, thermal behavior and luminescent characteristics of Eu^3+^-doped borosilicate glasses with compositions (35 − x/2)B_2_O_3_:(35 − x/2)SiO_2_:25La_2_O_3_:5CaO:0.5Eu_2_O_3_:xWO_3_, x = 0, 5, 10, and 15 mol% have been studied via X-ray and neutron diffraction (XRD, ND) combined with reverse Monte Carlo (RMC) modeling, infrared spectroscopy (IR), differential scanning calorimetry (DSC), photoluminescence (PL) spectroscopy and density measurements. IR and RMC structural modeling reveal that the glass network consists of SiO_4_ tetrahedra with non-bridging oxygen (Q^2^); diborate, pyroborate and metaborate units; [WO_4_]^2−^ and WO_6_ groups; and Si-O-W, Si-O-B, B-O-B and W-O-W linkages. Structural and density analyses show that incorporating up to 10 mol% WO_3_ increases structural disorder, depolymerizes the silicate matrix, and creates non-bridging oxygens (NBOs). In contrast, at 15 mol% WO_3_, the network exhibits crosslinking due to the development of interconnected, polymerized WO_6_ octahedra. Thermal investigations demonstrate exceptionally high glass transition temperatures (T_g_ > 700 °C) and notable thermal stability, as no crystallization occurred up to 800 °C in any sample. The slight decrease in T_g_ to 718 °C at 15 mol% WO_3_ validates network “softening” driven by the formation of polymerized WO_6_ octahedral structural units. Photoluminescence spectra showed a strong red emission at 612 nm from the ^5^D_0_→^7^F_2_ transition of Eu^3+^ under near-UV (392 nm) excitation. It was established that the addition of WO_3_ to borosilicate glasses leads to improvement of the emission properties of the active ion through structural modification. These results demonstrate that the optimized glass compositions hold potential for use as red phosphor materials.

## 1. Introduction

The development of luminescent rare earth (RE)-doped glasses has generated ongoing scientific interest because of their potential for large-scale production at lower cost, homogeneous light emission, high thermal stability, and the ability to incorporate a high concentration of RE ions. In particular, glasses doped with trivalent europium ions (Eu^3+^) are widely used as red-emitting materials for field emission technology and LEDs because of the strong red Eu^3+^ emission at 614 nm (^5^D_0_→^7^F_2_ transition). The luminescent properties of Eu^3+^ ions strongly depend on the chemical composition and structure of the host glass matrix [1,2]. Despite great successes, there are still unresolved problems around finding a chemically and thermally stable matrix for the active ion, achieving high-intensity fluorescence and high brightness, and the ability to achieve effective absorption in the ultraviolet region. That is why searching for a suitable Eu^3+^ glass matrix and obtaining a high-intensive pure red high-brightness luminescent product remains a challenge for scientists.

Among the many amorphous materials doped with rare earth ions, multicomponent borosilicate glasses have drawn considerable awareness and are used in various high-tech devices such as lasers [3], fiber-optic amplifiers [4] and photonic devices [5], as well as for luminescent materials [6]. They are distinguished by high chemical and thermal resistances, mechanical strength, high refractive index, low dispersion, low thermal expansion and high softening temperature. They are cost-effective and readily accessible. They are characterized by high transparency and high solubility of rare earth ions, which makes them suitable matrices for innovative optical applications [7]. Borosilicate glasses doped with Eu_2_O_3_ oxide are widely studied. The literature contains data on the preparation of such glasses in various systems: Bi_2_O_3_-SiO_2_-B_2_O_3_-Eu_2_O_3_; ZnO-SiO_2_-B_2_O_3_-Eu_2_O_3_; K_2_O-SiO_2_-B_2_O_3_-Eu_2_O_3_; Na_2_O-Gd_2_O_3_-B2O_3_-SiO_2_-Al_2_O_3_-Eu_2_O_3_; B_2_O_3_-SiO_2_-MgO-R-Eu_2_O_3_, [R = 10 (Li_2_O/Na_2_O/K_2_O)]; B_2_O_3_-SiO_2_-Al_2_O_3_-ZnO-Na_2_CO_3_-Eu_2_O_3_ and B_2_O_3_-SiO_2_-Y_2_O_3_-CaO-Eu_2_O_3_; and SiO_2_-B_2_O_3_-CaO-ReO (CeO_2_, Dy_2_O_3_, and Eu_2_O_3_) -Sb_2_O_3_ [8,9,10,11,12,13,14,15]. Their physical, optical and luminescent properties have been studied. These glasses exhibit red emission corresponding to the ^5^D_0_→^7^F_2_ transition (612 nm) in Eu^3+^ ions, making them promising materials for red light-emitting devices.

Incorporating transition metal oxides into rare earth-doped glasses has recently emerged as an effective method for tailoring their optical and structural properties. Tungsten trioxide (WO_3_) is particularly valuable in this role due to its high polarizability, high refractive index, and ability to alter glass network coordination [16,17,18,19,20,21]. The incorporation of WO_3_ generates additional non-bridging oxygens (NBOs) and increases structural disorder, which loosens the glass network and enhances the asymmetry of the local environment surrounding Eu^3+^ ions [17,20]. This asymmetry promotes forced electric dipole transitions, such as ^5^D_0_→^7^F_2_, thereby intensifying the characteristic red emission of Eu^3+^ (~612–613 nm). Furthermore, tungsten oxide acts as a sensitizer by non-radiatively transferring emission energy to the Eu^3+^ activator, further improving its luminescent properties [17]. Under conditions of high rare earth doping, the addition of WO_3_ helps prevent phase separation and stabilizes the amorphous network [18]. WO_3_-modified Eu^3+^-doped borosilicate glasses demonstrate high glass transition temperatures (exceeding 650 °C) and excellent resistance to crystallization, ensuring their durability for use in high-power optical devices [17]. All this mentioned above shows that investigating WO_3_-modified Eu^3+^-doped borosilicate glasses is important for obtaining an efficient red-emitting phosphor and solid-state lighting materials.

The objects of this work are the Eu^3+^-doped borosilicate glasses of the composition (35 − x/2)B_2_O_3_:(35 − x/2)SiO_2_:25La_2_O_3_:5CaO:0.5Eu_2_O_3_:xWO_3_, x = 0, 5, 10, and 15 mol%. The chosen compositions have been selected with our previous studies on similar glasses, that seem promising for use in optical technologies, in mind [17]. On the other hand, the borosilicate glasses with tungsten, which are doped with Eu^3+^ ions, are almost unexplored so far. There is only one recent paper in the literature regarding the WO_3_ incorporated Eu^3+^-doped lithium calcium borosilicate glasses (LCBS), demonstrating the beneficial roles of WO_3_ in enhancing structural asymmetry, modifying the optical band structure, and improving red emission efficiency [20]. Taking the above into account, the results obtained here will contribute to both the fundamental understanding of the structure–properties relationship of luminescent glass materials and the role of transition metals in tailoring their optical and structural properties, as well as to the preparation of novel glasses with possible applications in red-emitting photonic and optoelectronic devices.

The aim of this study is to obtain novel europium-doped borosilicate glasses containing different amount of WO_3_, to obtain information on their structure, thermal behavior and luminescence properties, and to identify the optimal WO_3_ concentration for glass performance optimization.

## 2. Materials and Methods

### 2.1. Sample Preparation

Glasses with the compositions in mol% of (35 − x/2)B_2_O_3_:(35 − x/2)SiO_2_:25La_2_O_3_:5CaO:0.5Eu_2_O_3_:xWO_3_, x = 0, 5, 10, and 15 mol% were prepared by the melt quenching method using reagent-grade La_2_O_3_, H_3_BO_3_, SiO_2_, CaCO_3_, ZnO, Eu_2_O_3_ and WO_3_ as raw materials. The homogenized batches (10 g) were melted at 1400 °C for 2 h in corundum crucibles in air. The melts were cast into preheated graphite molds to obtain bulk glass samples. Then, the glasses were transferred to a laboratory electric furnace, annealed at 500 °C for 1 h, and then cooled down to room temperature at a very slow cooling rate of about 0.5 °C/min.

### 2.2. Sample Characterization

The phase formation of the samples was established by X-ray phase analysis with a Bruker D8 Advance diffractometer, Karlsruhe, Germany using Cu Kα radiation in the 10 < 2θ < 60 range. The glass transition (T_g_) temperatures of the glasses were determined by differential scanning calorimetry (DSC) using a 404 Pegasus instrument, 2021, Netzsch, Selb, Germany, at a heating rate of 10 K/min in an Ar flow of 10 mL/s, using corundum crucibles with lids. The glass transition temperature (T_g_) was determined from the DSC curves using the onset method, via the extrapolated onset temperature, defined by the intersection of the baseline tangent before the transition and the tangent drawn through the region of maximum slope of the glass transition curve. The densities of the obtained glasses at room temperature were measured by the Archimedes principle using toluene (ρ = 0.867 g/cm^3^) as an immersion liquid on a Mettler Toledo electronic balance of sensitivity 10^−4^ g. From the experimentally evaluated density values, the molar volume (V_m_), the molar volume of oxygen (V_o_) (volume of glass in which 1 mol of oxygen is contained), and the oxygen packing density (OPD) of glasses obtained were estimated, using the following relations, respectively:

(1) $$V_{m}=\frac{\Sigma x_{i}M_{i}}{\rho_{g}}$$

(2) $$V_{o}=V_{m}\times \left(\frac{1}{\Sigma x_{i}n_{i}}\right)$$

(3) $$\mathrm{OPD}=1000\times C\times \left(\frac{\rho_{g}}{M}\right)$$

where x_i_ is the molar fraction of each component i, M_i_ is the molecular weight, ρ_g_ is the glass density, n_i_ is the number of oxygen atoms in each oxide, C is the number of oxygen atoms per formula units, and M is the total molecular weight of the glass compositions. The IR spectra of the obtained samples were measured using the KBr pellet technique on a Nicolet-320 FTIR spectrometer, Thermo Fisher Scientific, Madison, WI, USA with a resolution of ±4 cm^−1^, by collecting 64 scans in the range 1600–400 cm^−1^. A random error in the center of the IR bands was found as ±3 cm^−1^. Photoluminescence (PL) excitation and emission spectra at room temperature for all glasses were measured with a Spectrofluorometer FluoroLog3-22, 2014, Horiba JobinYvon, Longjumeau, France. Neutron diffraction studies of (35 − x/2)B_2_O_3_:(35 − x/2)SiO_2_:25La_2_O_3_:5CaO:0.5Eu_2_O_3_:xWO_3_, x = 0, 5, 10, and 15 mol% glasses were carried out in the momentum transfer range; Q-range: 0.45 to 9.8 Å-1 for 24 h using neutrons of de-Broglie wavelength, λ = 1.069 Å, at the 2-axis PSD diffractometer of Budapest Neutron Centre, Budapest, Hungary. For the neutron diffraction experiments, the glass samples (approximately 3.7–4.0 g) were pulverized and packed into 8 mm diameter thin-walled cylindrical vanadium containers. The raw neutron diffraction data were normalized using a vanadium standard and corrected for detector efficiency, background scattering, and absorption effects [22]. The total structure factor, S(Q), was subsequently derived using local software routines. To elucidate the short-range atomic structure, we processed the experimental S(Q) data using the RMC++ _new (2007) [23].

This reverse Monte Carlo method iteratively refines a 3D atomic configuration to minimize the squared difference between the simulated and experimental structure factors, based on the following equations:

(4) $$S(Q)=\sum_{i,j}^{k}w_{\mathrm{ij}}S_{\mathrm{ij}}(Q)$$

(5) $$S_{\mathrm{ij}}(Q)=1+\frac{4\pi \rho_{0}}{Q}\int_{0}^{r_{\max}}r\left[g_{\mathrm{ij}}(r)-1\right]\sin Q\;r\;\mathrm{dr}$$

(6) $$w_{\mathrm{ij}}=\frac{c_{i}c_{j}b_{i}b_{j}}{{\left[\sum_{i,j}^{k}c_{i}b_{j}\right]}^{2}}$$

In this expression, *c*_i_ and *c_j_* represent the molar fraction and the coherent neutron scattering length for atomic species *i*, respectively. *S_ij_*(*Q*) denotes the partial structure factors, and *w*_ij_ refers to the neutron scattering weight factors for the 21 unique atomic pairs found within the LaW series (given *k* = 6 components, which results in *k*(*k* + 1)/2 = 21 distinct pairs). The methodology employed during the RMC simulations to derive the partial atomic pair correlation functions, *g_ij_*(*r*), and the resulting coordination number distributions, is outlined below.

- The simulation was started with an initial random configuration by building a box that contained 10.000 atoms of La, Ca, Si, B, Eu, W and O with the atomic density, ρ_o_, values of 0.0736 Å^-3^, 0.0723 Å^-3^, 0.0713 Å^-3^ and 0.0712 Å^-3^ for the samples LaW0, LaW5, LaW10 and LaW15, respectively.
- The RMC model box lengths for the four samples were 25.70 Å, 25.85 Å, 25.97 Å and 25.99 Å for LaW0, LaW5, LaW10 and LaW15, respectively.

To prevent physically impossible atomic overlaps, the RMC simulations incorporated constraints on minimum interatomic distances, known as cut-off distances [24,25]. Multiple RMC iterations were conducted, with slight adjustments to these cut-off parameters; each run was meticulously validated to ensure the resulting partial pair correlation functions, *g_ij_*(*r*), and coordination number (CN_ij_) distributions were robust. For every sample, approximately fifty independent RMC configurations were generated, with each simulation involving more than 1.8 million accepted atomic moves.

## 3. Results and Discussion

### 3.1. XRD Data and Thermal Analysis

**Figure 1:**
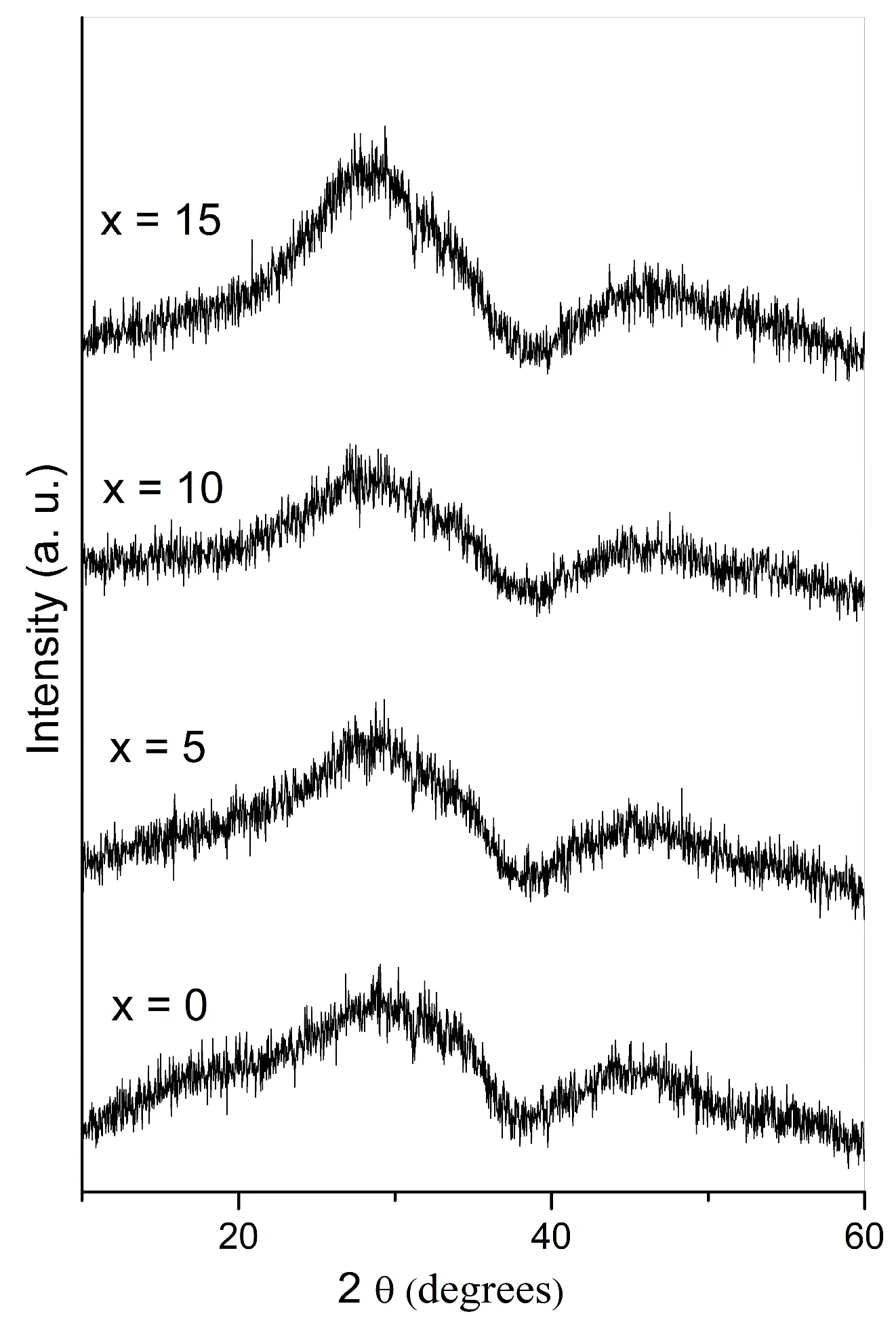
XRD patterns of (35 − x/2)B_2_O_3_:(35 − x/2)SiO_2_:25La_2_O_3_:5CaO:0.5Eu_2_O_3_: xWO_3_, x = 0, 5, 10, and 15 mol% glasses.

Analysis by means of X-ray diffraction provided confirmation that the materials that were obtained exhibited an amorphous nature. The characteristic diffraction patterns associated with the glasses that were produced are presented in Figure 1. Within every one of the diffraction profiles, a pronounced and diffuse broad scattering peak, commonly referred to as an amorphous halo, can be observed, and this peak reaches its maximum intensity within the 2θ range spanning from 25° to 35°. Throughout the measured window of 10° < 2θ < 60°, no sharp Bragg reflections can be detected, a fact that can be attributed to the lack of long-range atomic order. The modest sharpening of the diffraction hump that is evident in the diffractogram corresponding to the x = 15 glass implies that a decrease has taken place in the degree of structural disorder within the glass network that was formed [20].

**Figure 2:**
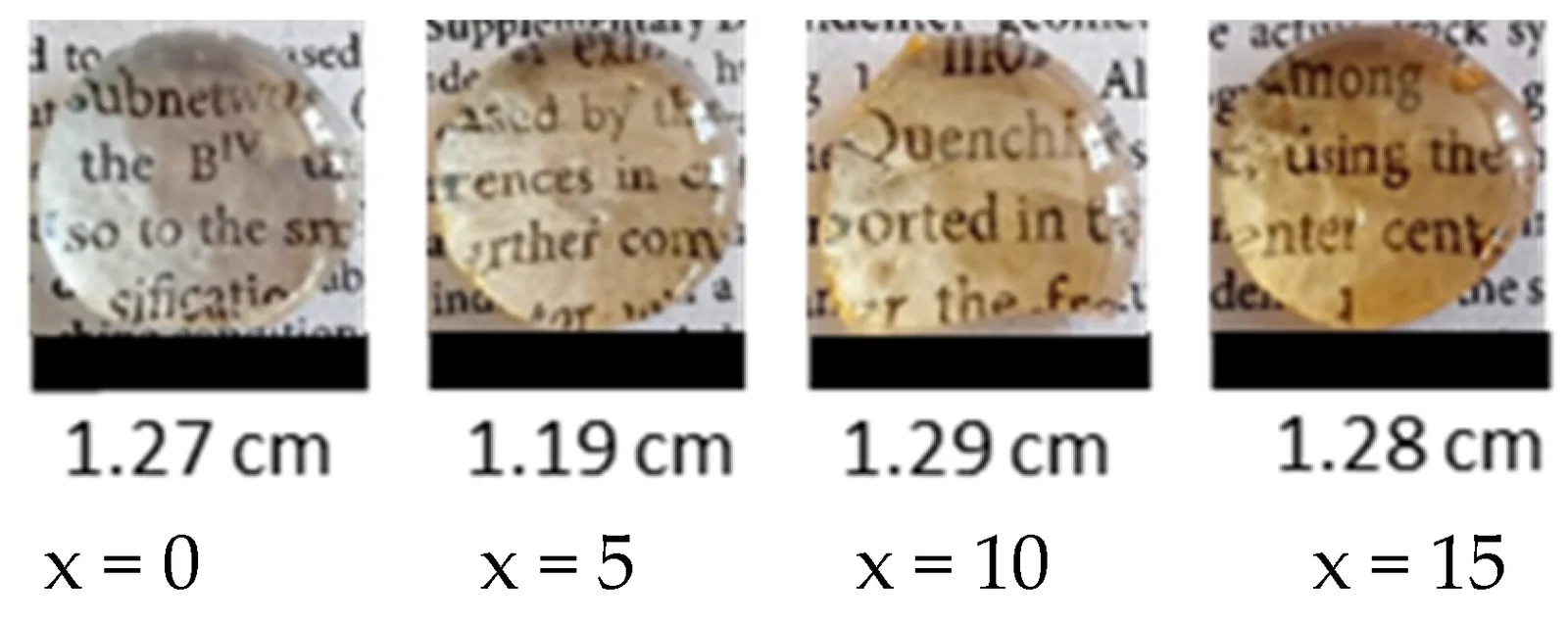
Photographs of (35 − x/2)B_2_O_3_:(35 − x/2)SiO_2_:25La_2_O_3_:5CaO:0.5Eu_2_O_3_:xWO_3_, x = 0, 5, 10, and 15 mol% glasses.

As demonstrated by the photographic images (Figure 2), transparent bulk glass samples were successfully produced.

**Figure 3:**
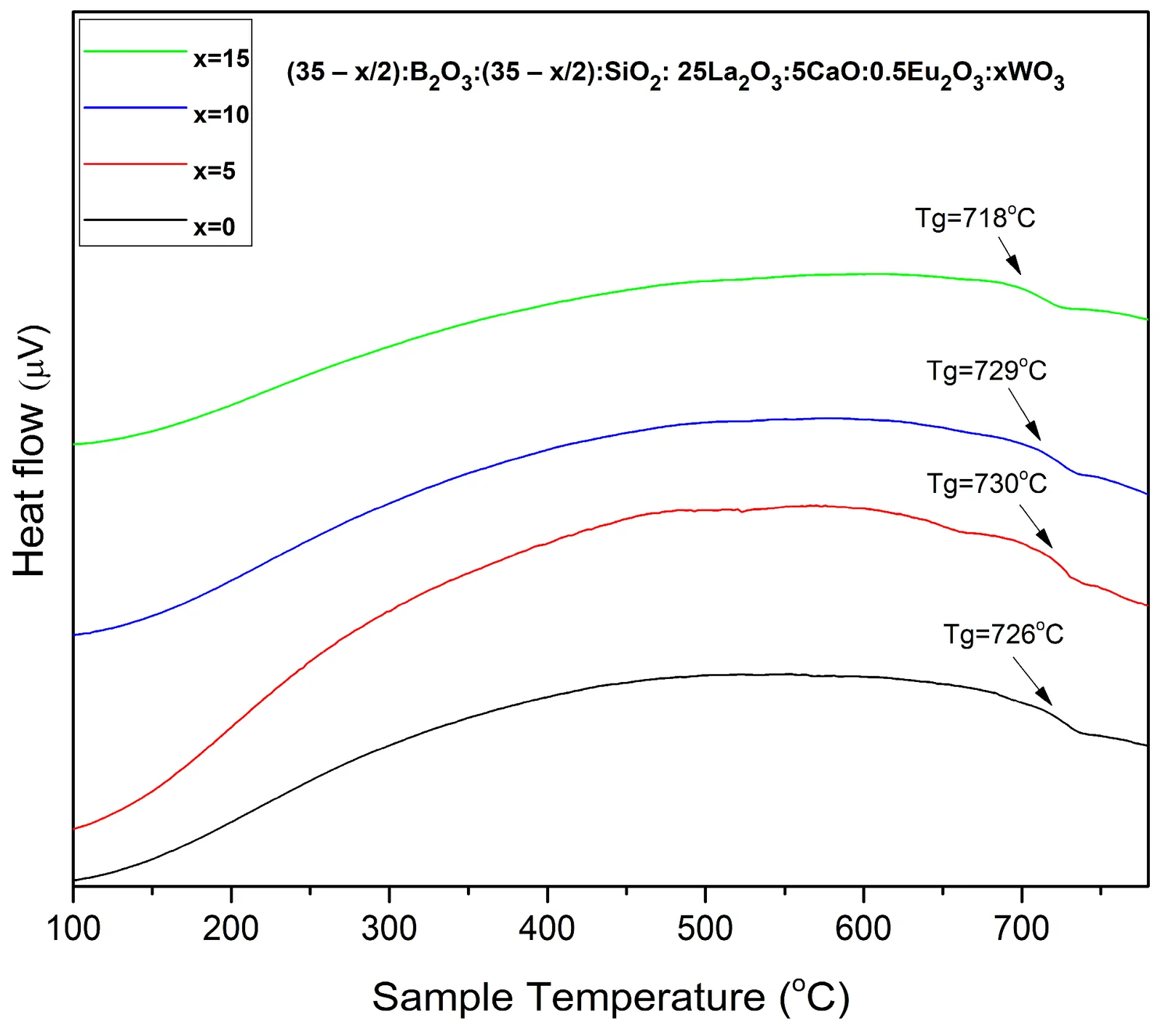
DSC curves of investigated glasses.

The thermal characteristics of the glasses that were synthesized in the present work are presented in Figure 3. Each of the curves obtained displays a hump, corresponding to the glass transition temperature, which is commonly denoted as T_g_. As is evident from the figure, these glasses are characterized by a notably high T_g_ value that exceeds 700 °C, a feature that proves highly advantageous when such glasses are employed in high-power optical devices. The value of T_g_ remains essentially unchanged as the composition is varied, which implies that the incorporation of WO_3_ exerts only minimal influence on the thermal parameters of the glasses examined in this study. Nevertheless, for the glass containing the maximum WO_3_ concentration of 15 mol % (x = 15), the value of T_g_ decreases slightly, falling to 718 °C. This reduction in T_g_ is consistent with the infrared and density analyses that will be discussed at a later point, since those analyses indicate that exceeding a threshold of 10 mol% WO_3_ gives rise to a structural transition in which WO_4_ tetrahedra are progressively replaced by more loosely linked, polymerized WO_6_ octahedra. As a consequence, the glass network undergoes a relative “softening”. For all of the glasses that were synthesized, no crystallization effects could be detected at temperatures up to 800 °C, thereby demonstrating that these glasses possess a high degree of thermal stability.

### 3.2. RMC Modeling and Results

**Figure 4:**
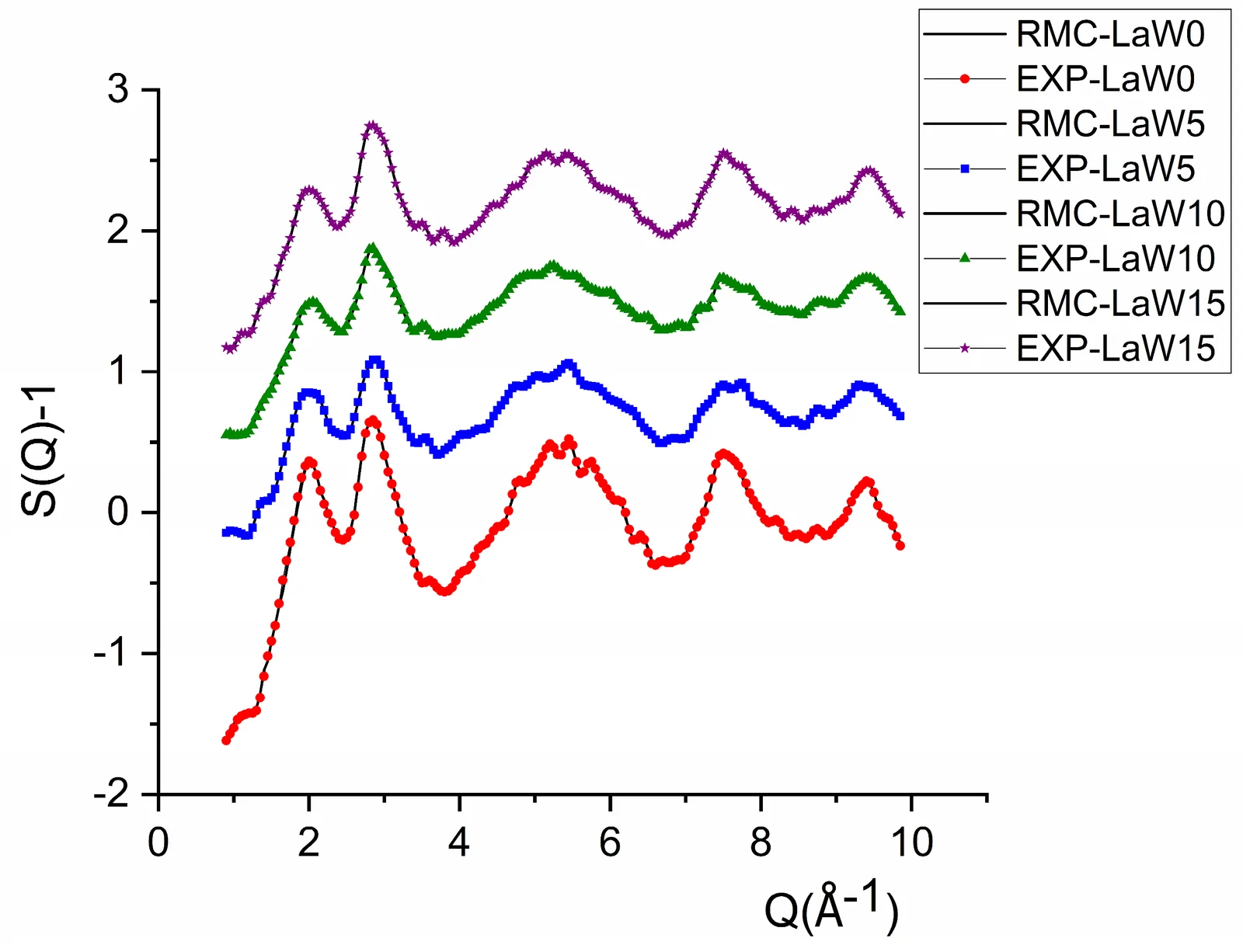
Experimental (color) and RMC (black line) simulated neutron scattering structure factors in LaW0–15 glasses.

**Figure 5:**
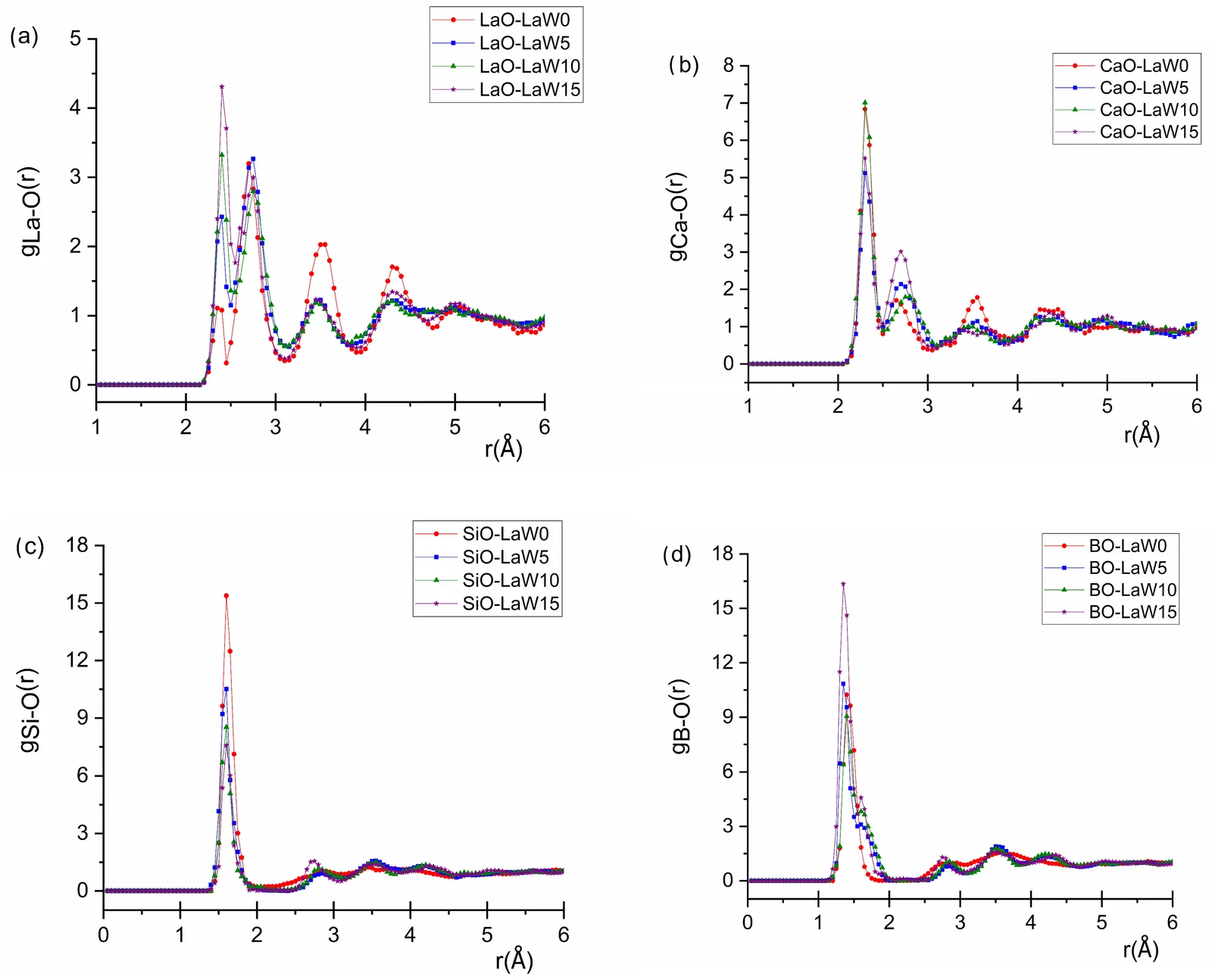

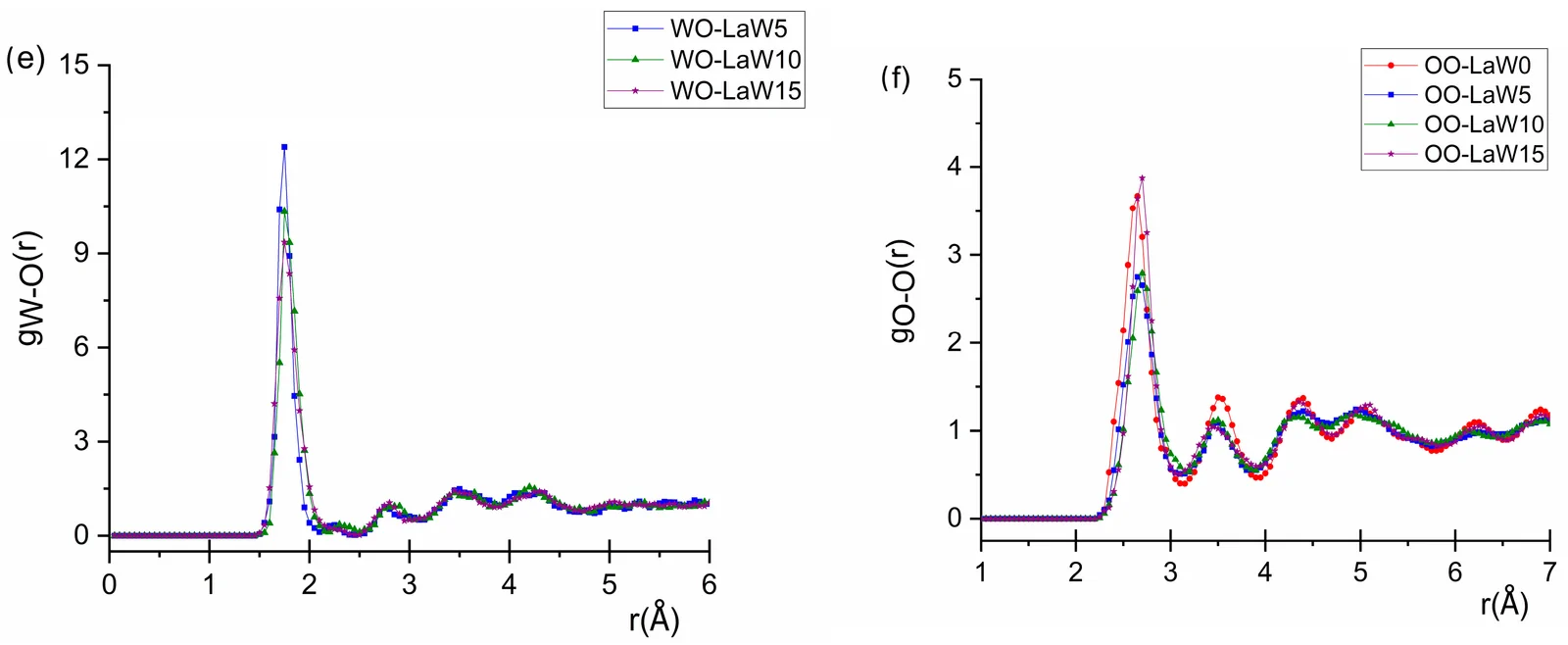
La-O (**a**), Ca-O (**b**), Si-O (**c**), B-O (**d**), W-O (**e**) and O-O (**f**) partial pair correlation functions of LaW0 (red), LaW5 (blue), LaW10 (green) and LaW15 (purple) glasses.

The RMC technique provided an excellent fit of the simulated structure factors (S(Q)-1) with the experimental one (Figure 4). From the RMC simulation, partial atomic pair correlation functions, g_ij_(r), and coordination number distributions, CNij, have been revealed with good stability and statistics. The La-O distribution functions show symmetrical peaks centered in the range of 2.37/2.40 ± 0.5 Å for all four samples [26,27]. The Ca-O distribution functions show peaks centered in the range of 2.30 ± 0.05 Å for all samples [28,29]. The Si-O distribution functions show characteristic peaks centered in the range of 1.60 ± 0.01 Å for all four samples [30,31,32]. The B-O distribution function shows a relatively broad first neighbor distance for the LaW0 sample at 1.40 ± 0.1 Å. For the other three samples with added WO_3_, in addition to the first peak at 1.35/1.40 Å, a shoulder appears at 1.6 ± 0.1 Å [33,34,35]. The Eu-O correlations show peaks at distances of 2.25 ± 0.5 Å. The nearest W-O distances show symmetric peaks at 1.75 ± 0.2 [36]. The O-O distances are at 2.65/2.75 ± 0.3 in the studied glasses. The first neighbor distances are presented in Figure 5 and Table 1. Within the limits of experimental uncertainties, the B-O nearest neighbor distances show slight changes with increasing WO_3_ mol % in the glass system. RMC modeling also enables the characterization of the second coordination sphere. The shortest second neighbor distances, obtained for the most interesting and important pairs, are Si-W, and Si-B, which supports the connection between the 4Si-O-3,4B units and serves as a reference for further data. Distances are presented in Table 2.

**Figure 6:**
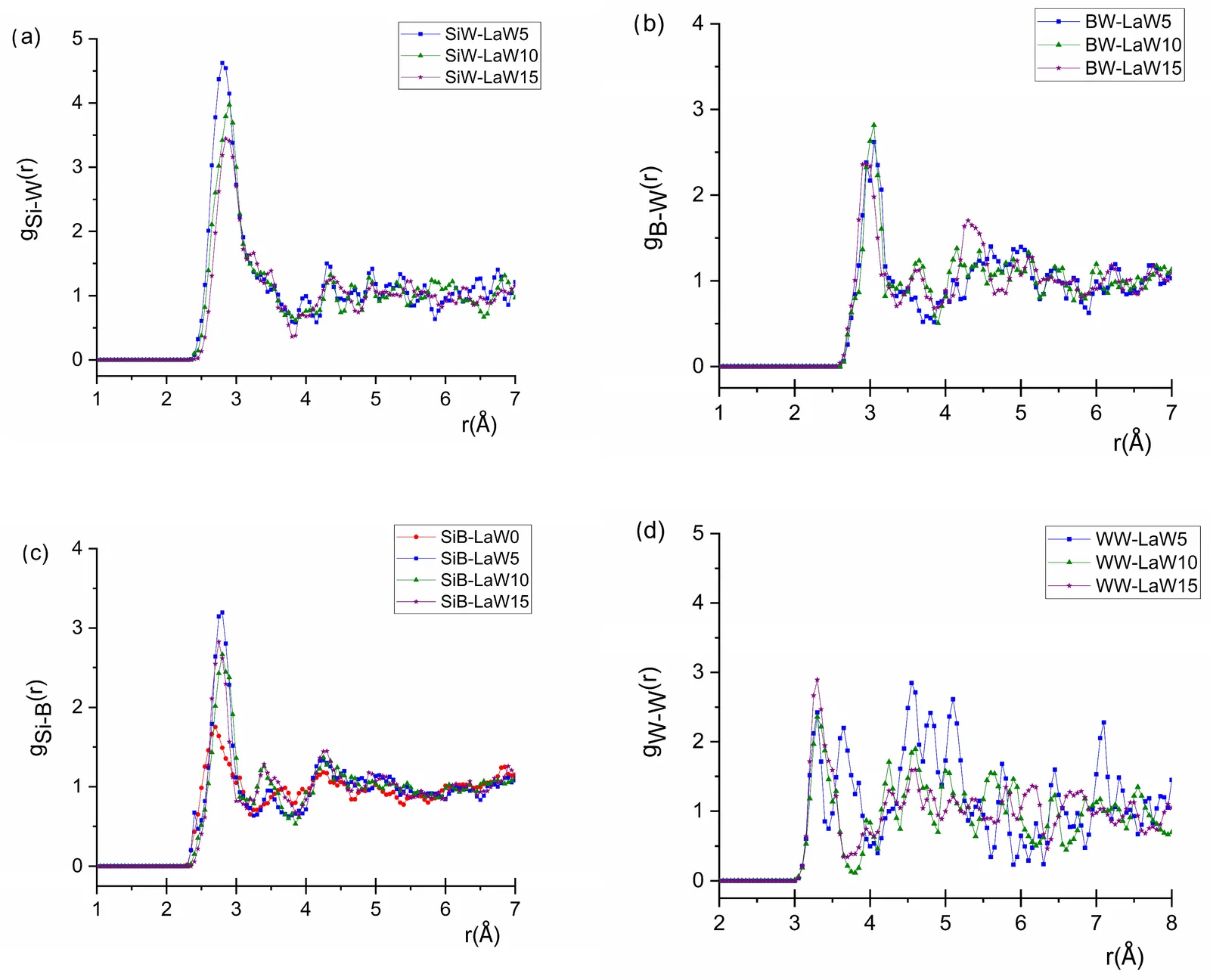
Si-W (**a**), B-W (**b**), Si-B (**c**) and W-W (**d**) second neighbor distances of LaW0 (red), LaW5 (blue), LaW10 (green) and LaW15 (purple) glasses.

These results indicate the existence of a pronounced intermediate-range ordering (Figure 6). Based on the interatomic distances and the coordination numbers, we predict that the glassy network structure is built up of SiO_4_ and WO_4_ tetrahedral units, along with trigonal BO_3_ and tetrahedral BO4 units. RMC calculations have revealed that the average coordination number of W is close to 4, within the limits of experimental uncertainty. Based on the diffraction data, we suppose—based on the W-O distances and the obtained coordination numbers—that W forms mainly tetrahedral units, and that the W-W distances (3.3 Å) reveal these units are connected, sharing common corners. These findings correlate with previous studies [36], which show that mostly WO_4_ units are present in glasses in lower tungsten oxide concentrations, even though the formation of WO_6_ is not excluded. These correlations could indicate the formations of 4W-O-4Si mixed linkages in the glass network. In the case of Eu-O coordination number, due to the very low Eu_2_O_3_ concentration, it is not possible to obtain reliable values.

The average coordination numbers obtained from the RMC analysis are presented in Table 3.

### 3.3. IR Spectra

**Figure 7:**
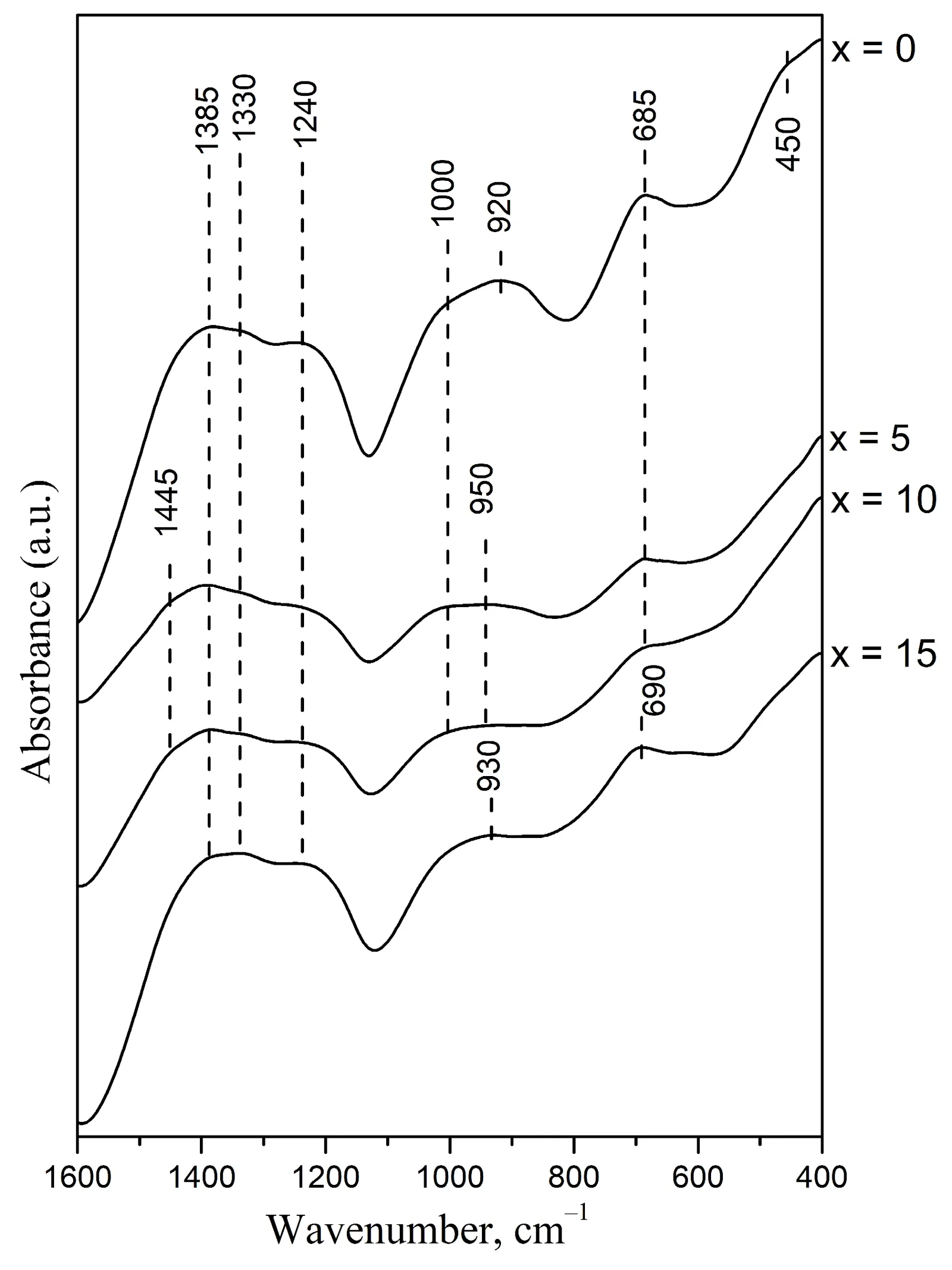
IR spectra of (35 − x/2)B_2_O_3_:(35 − x/2)SiO_2_:25La_2_O_3_:5CaO:0.5Eu_2_O_3_:xWO_3_, x = 0, 5, 10, and 15 mol% glasses.

IR spectroscopy was used to investigate the structural modifications resulting from the inclusion of tungsten in borosilicate glass. The normalized spectra are presented in Figure 7.

The IR spectrum of WO_3_-free glass (x = 0) exhibits two broad spectral features between 1600 and 1150 cm^−1^ and 1150–750 cm^−1^, a prominent band at 685 cm^−1^, and a shoulder at 450 cm^−1^. Absorption in the 1600–1150 cm^−1^ range is associated with the stretching vibration of the B-O bonds within trigonal BO_3_ units [19,37,38,39]. Specifically, the band at about 1385 cm^−1^ is attributed to the stretching vibration of non-bridging B-O^–^ bonds in metaborate triangular units BØ_2_O^−^ [19,39].

The band at 1330 cm^−1^ is connected with the B-O– stretch of pyroborate dimeric species [B_2_O_5_]^4−^ [40]. The band at 1240 cm^−1^ is due to the stretching vibration of BØ_3_ triangles involved in various type of superstructural borate groups (tri-, tetra- and pentaborates) [38,39,41]. On the other hand, this band could also originate from the BO_3_ stretching vibration of orthoborate units [38]. The spectral domain of 1150–750 cm^−1^ reflects superposed vibrations of silicate and borate groups with various bonding configurations. The shoulder observed at 1000 cm^−1^ is ascribed to the silicate Q^2^ tetrahedra (Q^n^ represents the SiO_4_ tetrahedra with a number of bridging oxygens (n)) and to the [BØ_4_]^−^ units from diborate groups [39,42]. The strong band centered at about 920 cm^−1^ is associated with the asymmetric B-O stretching vibrations of [BØ_4_]^−^ tetrahedra, as well as with the asymmetric stretching vibrations of mixed Si-O-B bonds [43]. The strong band at 685 cm^−1^ is due to the bending vibration of B-O-B bonds in superstructural units, containing BO_3_ and [BØ_4_]^−^ entities [39]. This band may also be due to the bending vibrations of mixed Si-O-B bridges [43]. The shoulder at 450 cm^−1^ represents bending modes of Si-O-Si linkages [43]. Additions of 5 and 10 mol% WO_3_ (Figure 6, x = 5 and x = 10) cause some changes in the vibrational spectra. As seen from the figure, the band intensities in the IR spectra of x = 5 and x = 10 glasses decrease compared with the intensities of the bands present in the spectrum of WO_3_-free glass (Figure 7, x = 0), evidencing increasing structural disorder with the introduction of WO_3_. A shoulder at 1445 cm^−1^ appears, which is due to asymmetric B-O^–^ stretching vibration of BO_3_ triangles containing non-bridging oxygens (NBOs) [19,39]. The absorption band in the 1150–750 cm^−1^ range becomes broader towards the lower wavenumber side of the spectra, due to the presence of the (WO_4_)^2−^ tetrahedra, whereas ν_3_ asymmetric stretching vibrations provide peaks in the wavenumber range 890–750 cm^−1^ [19]. The intensity of the band at 1000 cm^−1^ in the spectra of x = 5 and x = 10 glasses, due to SiO_4_ units with non-bridging oxygens (Q^2^), increases. The strong band at 920 cm^−1^, observed in the spectrum of the x = 0 glass (Figure 7; spectrum x = 0), upshifts to 950 cm^−1^, (Figure 7; spectra x = 5 and x = 10) because of the formation of mixed Si-O-W bonds, since IR spectra of silicate tungstates show bands at 950–1000 cm^−1^ range due the asymmetric stretching modes of the Si-O-W bridges [44,45]. The shoulder at 450 cm^−1^, present in the x = 0 glass associated with the vibrations of Si-O-Si linkages, disappears in the spectra of WO_3_-containing x = 5 and x = 10 glasses, evidencing that WO_3_ addition leads to the depolymerization of silicate network and increase in NBOs concentration. Formation of Si-O-W and disruption of Si-O-Si bonds evidence that WO_3_ incorporates successfully into the silicate network. The overall intensity of the IR spectrum of glass with 15 mol% WO_3_ (Figure 7; spectrum x = 15) increases, reflecting enhanced crosslinking in the glass network, as well as decreased degree of structural disorder [46]. In the IR spectrum of glass with 15 mol% WO_3_, the high-frequency shoulder at 1445 cm^−1^ disappears. For the glasses with x = 5 and x = 10, the band at 1445 cm^−1^ is ascribed to asymmetric B–O^−^ stretching vibrations of trigonal BO_3_ units containing non-bridging oxygens (NBOs). The fact that it is missing at x = 15 points is due to a marked drop in the concentrations of these borate groups. One may propose that the surplus modifier oxygen is structurally redistributed, causing the borate subnetworks to rearrange back into highly interconnected, superstructural configurations. The band at 950 cm^−1^, observed in the spectra of x = 5 and x =10 glasses, downshifts to 930 cm^−1^, while the band at 685 cm^−1^ slightly upshifts to 690 cm^−1^. Because of the complex character of the band at 950 cm^−1^, the red shift observed in the spectrum of x = 15 glass cannot be explained unambiguously. However, the slight upshift of the band 685 cm^−1^ to 690 cm^−1^, with increasing WO_3_ content up to 15 mol%, can be explained with the formation of polymerized WO_6_ octahedra in the network of the x = 15 glass. Asymmetric stretching vibrations of W-O-W linkages between WO_6_ octaherdra are found to provide a strong IR band at about 700 cm^−1^ in the spectra of glasses with high WO_3_ content [39]. The formation of interconnected WO_6_ octahedra indicates that WO_3_ has reached its solubility limit within a silicate glass network. The detailed assignments of the bands observed in the IR spectra of the present glasses are summarized in Table 4.

### 3.4. Density, Molar Volume, Oxygen Packing Density and Oxygen Molar Volume

Experimentally measured densities (ρ_g_) of the prepared glasses, along with the calculated values for molar volume (V_m_), oxygen molar volume (V_o_), and oxygen packing density (OPD), which provide insight into the structural modifications resulting from the addition of WO_3_ to the Eu^3+^-doped borosilicate glasses, are presented in Table 5.

As shown in Table 5, both the densities and molar volumes of the studied glasses increase as the WO_3_ concentration rises from 5 to 15 mol%. The density increment is attributed to an increase in the average molecular weight of the glasses, caused by the substitution of the lighter B_2_O_3_ (69.62 g/mol) and SiO_2_ (60.08 g/mol) with the significantly heavier WO_3_ (231.84 g/mol) [47]. Because molar volume is heavily influenced by the ionic radii of the constituent species, the upward trend in V_m_ is driven by the incorporation of W^6+^ ions. These ions have a larger ionic radius (0.60 Å) compared to B^3+^ (0.23 Å) and Si^4+^ (0.26 Å), which creates excess free volume and consequently increases the total molar volume of the glasses [48,49,50]. The oxygen molar volume (V_O_) and oxygen packing density (OPD) provide insight into the arrangement of oxygen ions within the glass structure [51]. Generally, a more tightly connected network is characterized by lower V_O_ and higher OPD values. In glasses containing up to 10 mol% WO_3_ (samples x = 5 and x = 10), V_O_ increases and OPD decreases relative to the WO_3_-free glass (x = 0). This suggests that the additions of 5 and 10 mol % WO_3_ increase the concentration of non-bridging oxygens (NBOs), leading to a less interconnected and more disordered network. Glass with higher WO_3_ concentration of 15 mol% (sample x = 15) exhibits lower V_O_ and higher OPD as compared with the values of those parameters of x = 5 and x = 10 glasses. Based on the present IR data, this is attributed to enhanced crosslinking in the glass network and the formation of bridging W–O–W bonds, resulting from the presence of polymerized WO_6_ units.

### 3.5. Photoluminescent Properties

**Figure 8:**
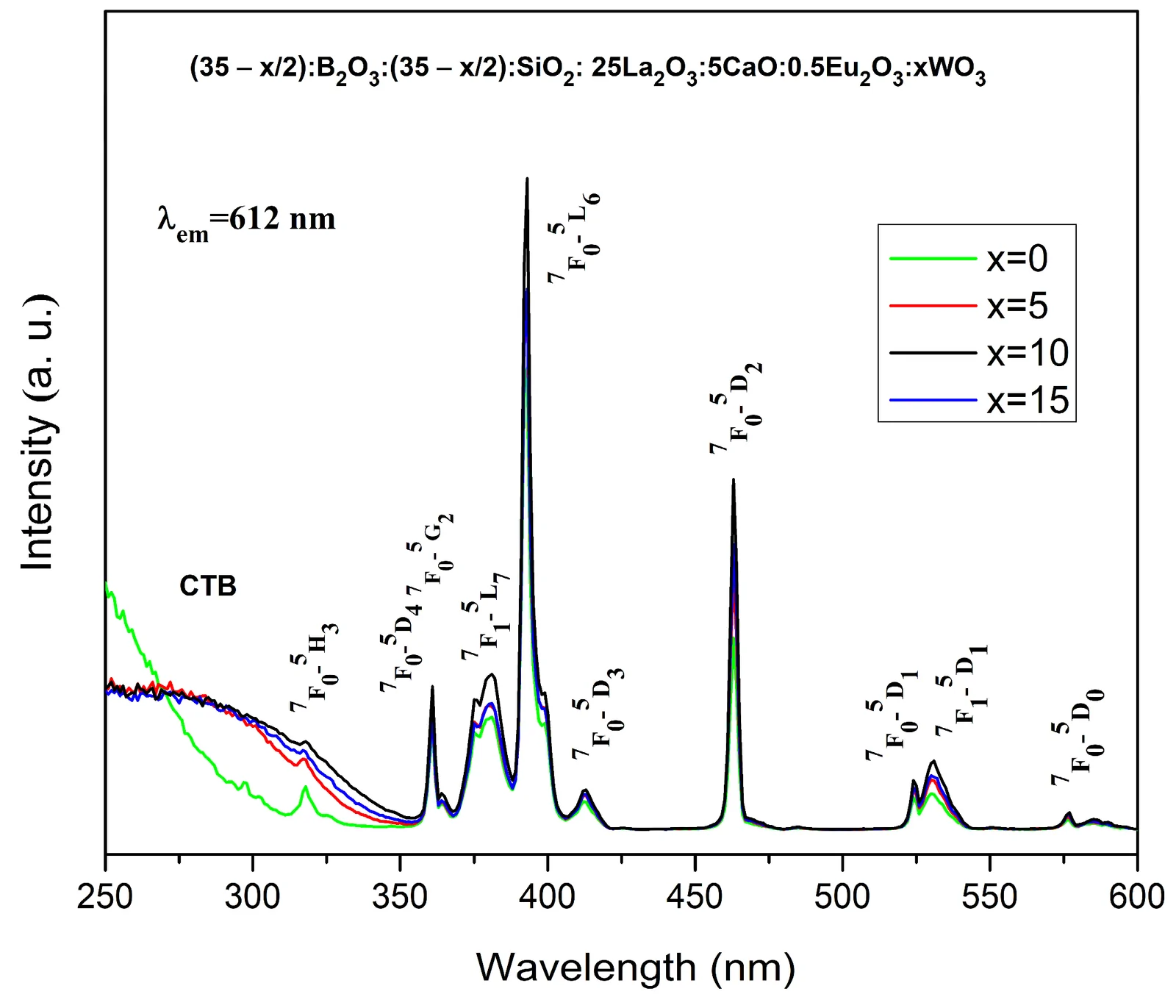
Excitation spectra of (35 − x/2)B_2_O_3_:(35 − x/2)SiO_2_:25La_2_O_3_:5CaO:0.5Eu_2_O_3_:xWO_3_, x = 0, 5, 10, 15 mol% glasses.

The excitation spectra (Figure 8) of the synthesized glasses, monitored at the Eu^3+^ emission wavelength of 612 nm (^5^D_0_→^7^F_2_ transition of Eu^3+^ ions) exhibit a broad excitation band in the ultraviolet region below 350 nm, along with several sharp 4f transitions of Eu^3+^ located at 317 nm (^7^F_0_→^5^H_3_), 360 nm (^7^F_0_→^5^D_4_), 375 nm (^7^F_0_→^5^G_2_), 381 nm (^7^F_1_→^5^L_7_), 393 nm (^7^F_0_→^5^L_6_), 412 nm (^7^F_0_→^5^D_3_), 463 nm (^7^F_0_→^5^D_2_) 524 (^7^F_0_→^5^D_1_), 531 nm (^7^F_1_→^5^D_1_) and 577nm (^7^F_0_→^5^D_0_) [2].

**Figure 9:**
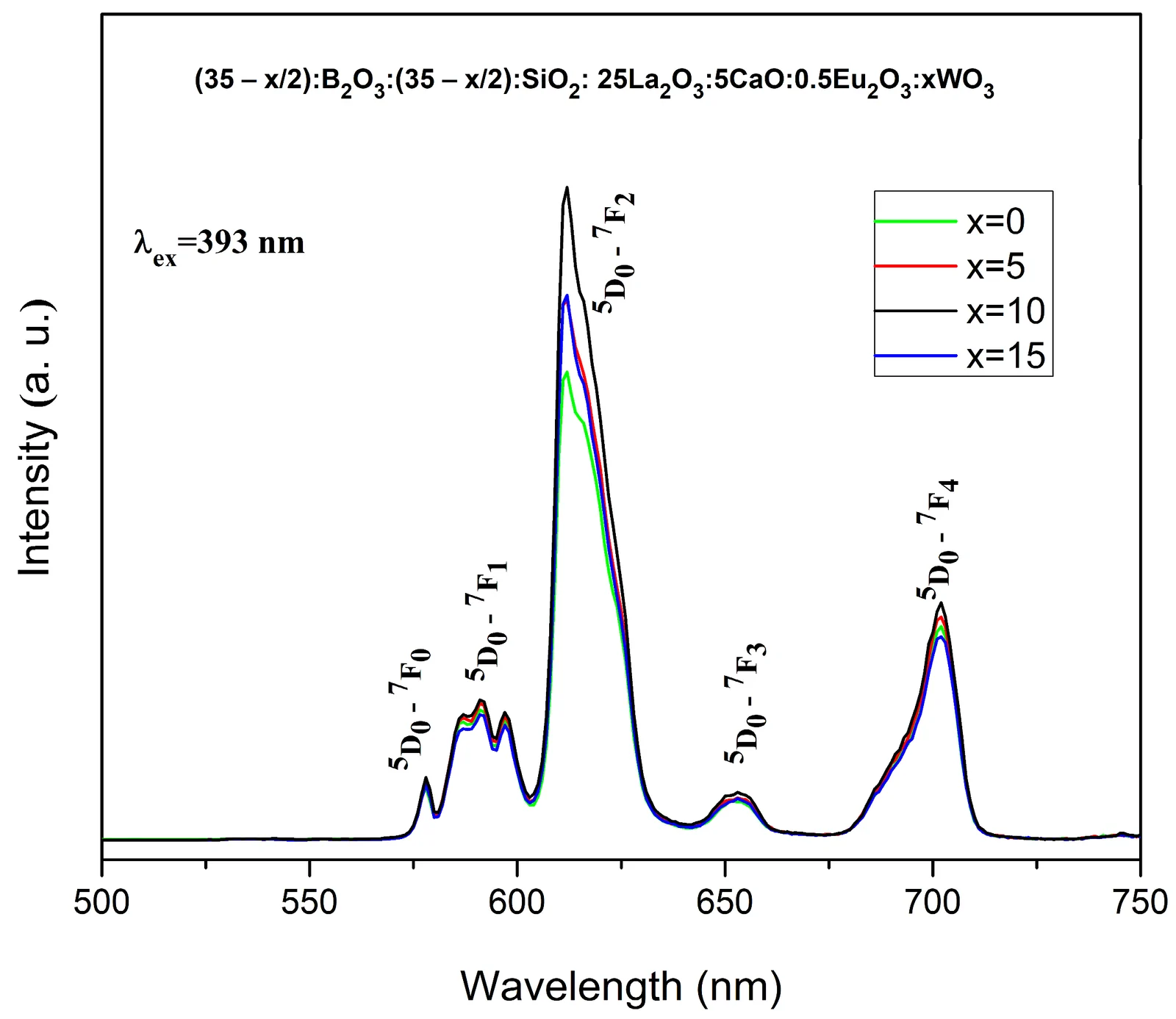
Emission spectra of (35 − x/2) B_2_O_3_:(35 − x/2) SiO_2_:25La_2_O_3_:5CaO:0.5Eu_2_O_3_:xWO_3_, x = 0, 5, 10, 15 mol% glasses.

Among the observed excitation lines, the transition at 393 nm exhibits the highest intensity and was therefore chosen as the excitation wavelength for the emission measurements. The second most prominent band is located at 463 nm. Both spectral positions fall within the operating ranges of near-UV LEDs (250–400 nm) and blue-emitting LED chips (430–470 nm), which is very advantageous for achieving good excitation of the synthesized materials, since Eu^3+^ 4f transitions generally exhibit relatively low intensities because they are formally forbidden by the Laporte selection rule [52]. The broad UV band in the UV region is assigned to charge-transfer transitions of Eu^3+^ (O^2−^→Eu^3+^) [53,54,55,56] and O^2−^→W^6+^ in the WO_n_ host absorbing groups (WO_n_ = WO_4_ and WO_6_) [53,57,58]. Owing to the considerable spectral overlap of these contributions, their individual assignments cannot be distinguished. Figure 9 shows the photoluminescence emission spectra of Eu^3+^-doped borosilicate glasses. The spectra were recorded under 393 nm wavelength of excitation. The emission spectra (Figure 9) present the characteristic visible radiative transitions of Eu^3+^ ions from the ^5^D_0_ excited state to the lower-lying ^7^F_0_, ^7^F_1_, ^7^F_2_, ^7^F_3_, ^7^F_4_ ground states, observed at 575 nm, 591 nm,612 nm, 652 nm and 702 nm [2]. A pronounced red band centered at 612 nm dominates the emission spectra.

The glass with 10 mol % WO_3_ exhibits the strongest emission. By comparison, the glass containing 15 mol% WO_3_ shows decreased emission intensity. The most intensive emission band, located in the red region at 612 nm, is attributed to the electric dipole ^5^D_0_→^7^F_2_ transition, which is strongly influenced by the local crystal field environment surrounding the Eu^3+^ ions, while ^5^D_0_→^7^F_1_ is a magnetic dipole transition, not affected by the local symmetry [2,53]. The predominance of the electric dipole transition over the magnetic dipole one indicates that the Eu^3+^ ions are predominantly located in low-symmetry, non-centrosymmetric sites within the glass network. The ratio of ^5^D_0_→^7^F_2_ and ^5^D_0_→^7^F_1_ transitions, referred to as red-to-orange intensity asymmetry ratio R = (^5^D_0_→^7^F_2_)/(^5^D_0_→^7^F_1_), provides information about the degree of local symmetry in the glass matrix, as well as the strength of Eu–O covalence in various Eu^3+^-doped materials [59,60]. Low values of this ratio are connected with a more symmetric local environment around the Eu^3+^ ions, a lower degree of Eu–O covalency, and lower emission intensity, while an increase in the R ratio indicates enhanced asymmetry of the Eu^3+^ coordination environment, as well as stronger covalent interactions between Eu–O, which can favor stronger emission and, hence, an improved optical performance. Generally, it is accepted that ratio values greater than 1 reflect the predominance of the hypersensitive ^5^D_0_→^7^F_2_ electric dipole transition and correspond to the locations of Eu^3+^ ions in sites with relatively lower symmetry, while values below 1 indicate a relatively stronger magnetic dipole contribution and location of the active ion in high-symmetry sites. The samples exhibiting the lowest R values provide the most symmetric local coordination environments for the Eu_3+_ ions within the investigated glass series.

The calculated R values of the prepared glasses are summarized in Table 6.

An increase in asymmetric ratio is observed with inclusion and rise in the WO_3_ content in the glass compositions up to 10 mol% of tungsten. The calculated ratios for the present glasses are higher than the values observed for borosilicate glasses previously synthesized by us [17], and from other borosilicate glasses reported in the literature [9,11,42,63,64,65,66], and have comparable values with zinc–borate glass compositions previously prepared by us [18,24], as well as with the commercial phosphor material [67,68]. The observed high values of R indicate that the Eu^3+^ ions are predominantly accommodated in low-symmetry sites, suggesting an enhanced covalent character of the Eu–O bonds. As was deduced from the IR structural data, as well as the physical parameters evaluated, the introduction of WO_3_ up to 10 mol% into the borosilicate network disrupts the rigid network, creating non-bridging oxygens and distorting the local coordination sphere around Eu^3+^ ions, significantly lowering their local symmetry and strengthening the covalent character of Eu-O bonds, thereby increasing the electron–dipole transition probability. The highest R value belongs to the composition with 10 mol% WO_3_, which also exhibits the strongest emission intensities. The decrease in the emission intensity for glass composition with 15 mol% WO_3_ is consistent with lowering the asymmetry ratio due to an increase in the site symmetry of the Eu^3+^ ion.

**Figure 10:**
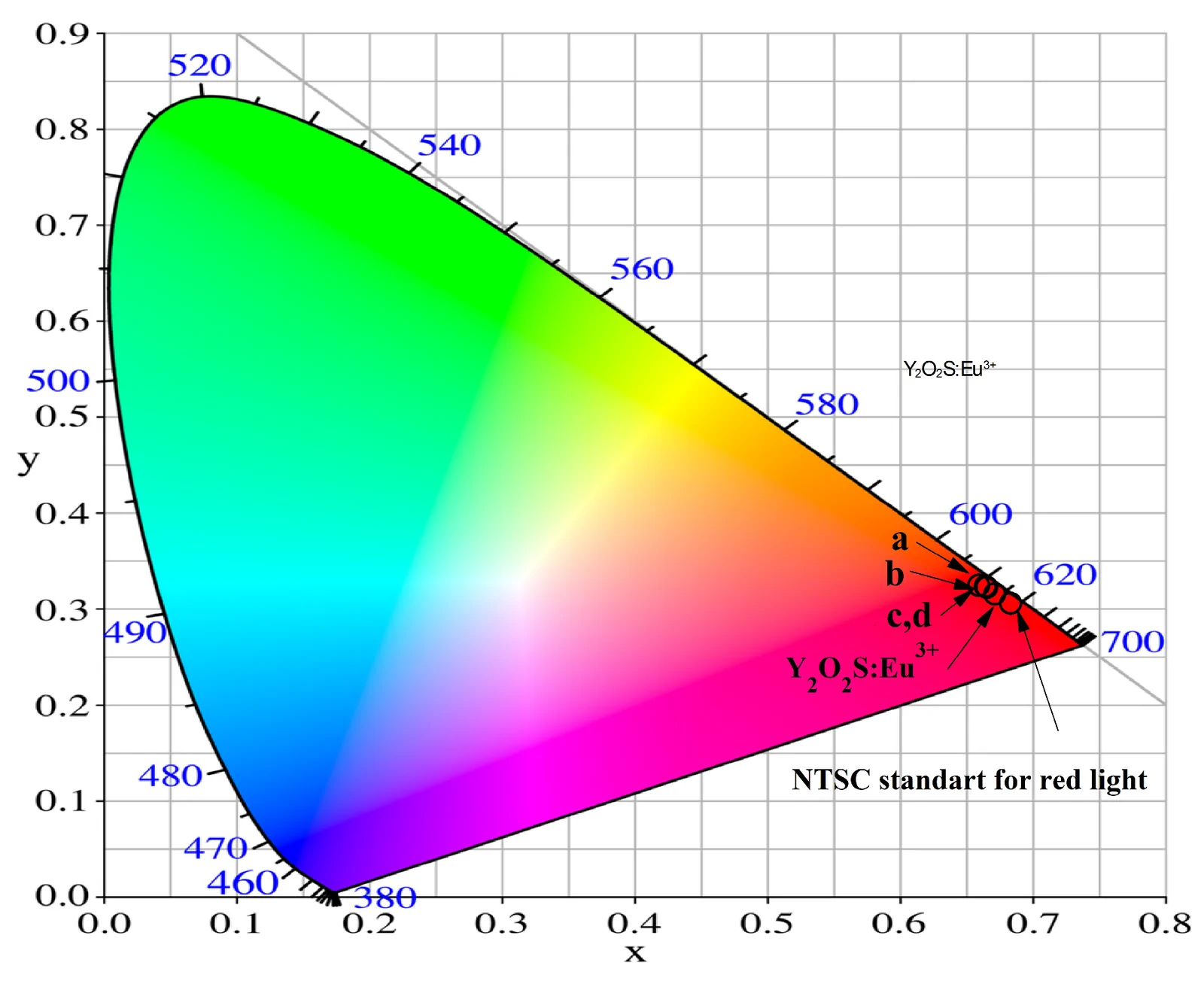
CIE chromaticity diagram of (35 − x/2)B_2_O_3_:(35 − x/2)SiO_2_:25La_2_O_3_:5CaO:0.5Eu_2_O_3_:xWO_3_, investigated glasses: (**a**) x = 0, (**b**) x = 5, (**c**) x = 10, (**d**) x = 15 mol%.

To further characterize the luminescence characteristics and the emission colors of the prepared glasses, the Commission Internationale de l’Éclairage (CIE) 1931 chromaticity diagram was used [69]. The CIE chromaticity coordinates of the synthesized borosilicate glasses were derived from the respective emission spectra presented in Figure 10 using SpectraChroma software (version 1.0.1, CIE Coordinate Calculator) [70].

As presented in Figure 10, the calculated coordinates are very similar for all investigated compositions and are situated in the red region of the CIE chromaticity diagram. The corresponding values are shown in Table 7. The obtained coordinates are close to both the NTSC standard chromaticity coordinates for red light (0.670, 0.330) and those reported for the commercial red-emitting phosphor Y_2_O_2_S:Eu^3+^ (0.658, 0.340) [71].

Furthermore, the chromatic characteristics of the red emission from the Eu^3+^-activated borosilicate glasses can be studied by calculating the color-correlated temperature (CCT) by the McCamy empirical formula [72]:

CCT = −449n^3^+ 3525n^2^ − 6823.3n + 5520.33 (7)

where n = (x − xe)/(y − ye) is the reciprocal slope, (xe = 0.332, ye = 0.186) represent the epicenter of convergence, and x and y are the chromaticity coordinates. The light sources with CCT values below 3200 K are generally classified as warm, whereas those exceeding 4000 K are regarded as cool light sources [48]. The calculated CCT values of the Eu^3+^-doped glasses range from 2247.28 K to 2363.19 K (Table 2), indicating that the investigated compositions exhibit warm-toned red emission and may be considered promising red-emitting materials for solid-state lighting applications.

## 4. Conclusions

This work evaluates the structural, thermal, and luminescent variations induced by incorporating WO_3_ into Eu^3+^-doped borosilicate glasses. IR spectroscopy, density measurements, and RMC modeling demonstrate that, up to 10 mol%, WO_3_ breaks down the rigid network connectivity and elevates the concentration of non-bridging oxygens, whereas exceeding this threshold (15 mol%) induces a network rearrangement characterized by increased crosslinking and the polymerization of WO_6_ octahedra. Thermal analysis shows that these glasses have high glass transition temperatures (above 700 °C) and can resist crystallization up to 800 °C, making them suitable for use in high-power optical systems. The addition of WO_3_ increases structural disorder and boosts both the local asymmetry and the covalent nature of the Eu-O bonds, which results in stronger luminescence from the Eu^3+^ ions. The photoluminescence spectra of these glasses show strong red emission at 612 nm and a very high luminescence asymmetry ratio R (above 5), both of which suggest that the Eu^3+^ ions are located in distorted, low-symmetry sites within the glass structure. It was established that the glass containing 10 mol% WO_3_ exhibits the strongest red emission due to maximized local distortion, representing the optimum composition for potential visible red-emitting applications.

## Figures and Tables

**Table 1:** X-O interatomic distances, g_ij_(Å) obtained from the RMC simulation.

	*g*_La-O_(*r*)	*g*_Ca-O_(*r*)	*g*_Si-O_(*r*)	*g*_B-O_(*r*)	*g*_Eu-O_(*r*)	*g*_W-O_(*r*)	*g*_O-O_(*r*)
**LaW0**	2.37/2.70/3.55 ± 0.1	2.30/2.65 ± 0.1	1.60 ± 0.05	1.40 ± 0.05	2.20 ± 0.5	-	2.65/3.50 ± 0.3
**LaW5**	2.40/2.75 ± 0.1	2.30/2.70 ± 0.1	1.60 ± 0.05	1.35/(1.60) ± 0.05	2.25 ± 0.5	1.75 ± 0.1	2.65/3.50 ± 0.3
**LaW10**	2.40/2.75 ± 0.1	2.30/2.80 ± 0.1	1.60 ± 0.05	1.40/(1.60) ± 0.05	2.25 ± 0.5	1.75 ± 0.1	2.70/3.50 ± 0.3
**LaW15**	2.40/2.75 ± 0.1	2.30/2.70 ± 0.1	1.60 ± 0.05	1.35/(1.60) ± 0.05	2.25 ± 0.5	1.75 ± 0.1	2.70/3.50 ± 0.3

**Table 2:** Second neighbor distance for the W-distances, obtained from the RMC simulation.

	*g*_Si-W_(*r*)	*g*_Si-B_(*r*)	*g*_W-W_(*r*)
**LaW0**	-	2.70 ± 0.5	-
**LaW5**	2.80 ± 0.5	2.80 ± 0.5	3.30/(3.65) ± 3
**LaW10**	2.90 ± 0.5	2.80 ± 0.5	3.30 ± 1
**LaW15**	2.90 ± 0.5	2.75 ± 0.5	3.30 ± 1

**Table 3:** The average coordination numbers (*CN*ij) were derived from the RMC simulation results, with the specific integration intervals (r_min_ to r_max_) used for these calculations provided in parentheses.

	LaW0	LaW5	LaW5	LaW5
***CN*** **_La-O_**	4.55 ± 0.2 (r_min_: 2.2–r_max_: 3.0)	5.68 ± 0.2 (r_min_: 2.2–r_max_: 3.05)	6.05 ± 0.2 (r_min_: 2.2–r_max_: 3.1)	6.20 ± 0.2 (r_min_: 2.2–r_max_: 3.05)
***CN*** **_Ca-O_**	3.15 ± 0.1 (r_min_: 2.05–r_max_: 2.5)	3.09 ± 0.1 (r_min_: 2.1–r_max_: 2.5)	3.05 ± 0.1 (r_min_: 2.1–r_max_: 2.55)	3.02 ± 0.1 (r_min_: 2.1–r_max_: 2.5)
***CN*** **_Si-O_**	3.85 ± 0.05 (r_min_: 1.4–r_max_: 1.95)	3.91 ± 0.05 (r_min_: 1.4–r_max_: 1.9)	3.95 ± 0.05 (r_min_: 1.4–r_max_: 1.9)	3.96 ± 0.05 (r_min_: 1.35–r_max_: 1.95)
***CN*** **_B-O_**	2.98 ± 0.05 (r_min_: 1.2–r_max_: 1.8)	3.20 ± 0.05 (r_min_: 1.2–r_max_: 1.95)	3.34 ± 0.05 (r_min_: 1.2–r_max_: 1.95)	3.45 ± 0.05 (r_min_: 1.2–r_max_: 1.95)
***CN*** **_W-O_**	–	3.83 ± 0.1 (r_min_: 1.55–r_max_: 2.1)	3.90 ± 0.1 (r_min_: 1.55–r_max_: 2.2)	3.92 ± 0.1 (r_min_: 1.5–r_max_: 2.15)
***CN*** **_O-O_**	5.86 ± 0.1 (r_min_: 2.3–r_max_: 3.0)	5.76 ± 0.1 (r_min_: 2.3–r_max_: 3.15)	5.64 ± 0.1 (r_min_: 2.25–r_max_: 3.15)	5.58 ± 0.1 (r_min_: 2.25–r_max_: 3.1)

**Table 4:** Infrared bands (in cm^−1^) and their assignments for (35 − x/2)B_2_O_3_:(35 − x/2)SiO_2_:25La_2_O_3_:5CaO:0.5Eu_2_O_3_:xWO_3_, x = 0, 5, 10, and 15 mol% glasses.

Peak Position, cm^−1^				Band Assignment	Ref.
x = 0	x = 5	x = 10	x = 15		
450	-	-	-	Bending modes of Si-O-Si linkages	[43]
685	685	685	690	Bending vibration of B-O-B bonds in superstructural units, containing BO_3_ and [BØ_4_]^−^ entities	[39]
920	950	950	930	ν_as_ B-O of [BØ_4_]^−^; ν_as_ Si-O-B bonds	[43]
1000	1000	1000	1000	Q^2^ tetrahedra; [BØ_4_]^−^ units from diborate groups	[39,42]
1240	1240	1240	1240	ν BØ_3_ in superstructural borate groups (tri-, tetra- and pentaborates)	[38,39,41]
1330	1330	1330	1330	B-O^–^ stretch of pyroborate dimeric species, [B_2_O_5_]^4−^	[40]
1385	1385	1385	1385	ν B–O^−^ stretch in BØ_2_O^−^ units	[19,39]
-	1445	1445	-	ν_as_ B–O^−^ stretch of BO_3_	[19,39]

**Table 5:** Physical parameters: density (ρ_g_), molar volume (V_m_), oxygen molar volume (V_o_), oxygen packing density (OPD) of (35 − x/2)B_2O3_:(35 − x/2)_SiO2_:25La_2_O_3_:5CaO:0.5Eu_2_O_3_:xWO_3_, x = 0, 5, 10, and 15 mol% glasses.

Sample ID	ρ_g_ (g/cm^3^)	V_m_ (cm^3^/mol)	V_o_ (cm^3^/mol)	OPD (g atom/L)
x = 0	3.838 ± 0.003	34.24	13.32	75.06
x = 5	4.009 ± 0.005	34.86	13.46	74.30
x = 10	4.192 ± 0.007	35.33	13.48	74.16
x = 15	4.426 ± 0.002	35.35	13.39	74.68

**Table 6:** Comparison of intensity ratio R of studied glasses with different host matrices.

Glass Composition	Relative Intensity Ratio, R	Reference
35B_2_O_3_:35SiO_2_:25La_2_O_3_:5CaO:0.5Eu_2_O_3_	4.59	Current work
32.5B_2_O_3_:32.5SiO_2_:25La_2_O_3_:5CaO:0.5Eu_2_O_3_:5WO_3_	4.89	Current work
30B_2_O_3_:30SiO_2_:25La_2_O_3_:5CaO:0.5Eu_2_O_3_:10WO_3_	5.71	Current work
27.5B_2_O_3_:27.5SiO_2_:25La_2_O_3_:5CaO:0.5Eu_2_O_3_:15WO_3_	5.41	Current work
(52.5 − x/2) B_2_O_3_: (12.5 − x/2) SiO_2_:25La_2_O_3_:5ZnO:5CaO:0.5Eu_2_O_3_:x WO_3_, x = 0, 2.5, 5, 7.5, 10, 20	4.12–4.92	[17]
50ZnO:(49 − x)B_2_O_3_:1Bi_2_O_3_:xWO_3_:0.5Eu_2_O_3_ x = 1, 5, 10,	4.61–5.73	[39]
50ZnO:(50 − x)B_2_O_3_:xNb_2_O_5_:0.5Eu_2_O_3_:, x = 0, 1, 3 and 5 mol%	4.31–5.16	[61]
50ZnO:(50 − x)B_2_O_3_:0.5Eu_2_O_3_:xWO_3_, x = 0, 1, 3, 5.	4.34–5.57	[62]
35B_2_O_3_–20SiO_2_-(15 − x) Al_2_O_3_–15ZnO–15Na_2_CO_3_-xEu_2_O_3_, x = 0, 0.5, 1.0, 1.5, 2.0, 2.5	3.62–3.92	[11]
60SiO_2_-15B_2_O_3_-15Na_2_O-8CaF_2_-2NaF-0.25Eu_2_O_3_	2.74	[63]
50B_2_O_3_-19SiO_2_-20Na_2_O-10CaO-1Eu_2_O_3_	3.151	[64]
50B_2_O_3_-14SiO_2_-20Na_2_O-10CaO-5ZnO-1Eu_2_O_3_	3.352	
50B_2_O_3_-14SiO_2_-20Na_2_O-10CaO-5TeO_2_-1Eu_2_O_3_	4.269	
74.5 B_2_O_3_ + 10SiO_2_ + 5 MgO + R + 0.5 Eu_2_O_3_ [where R = 10 (LiO_2_/Na_2_O/K_2_O) for series A and R = 5 (Li_2_O + Na_2_O/Li_2_O + K_2_O/K_2_O + Na_2_O)	2.093–2.269	[9]
20SiO_2_-(20 − x)B_2_O_3_–10Al_2_O_3_–10ZnO–30NaF–10ZnF_2_-xEu_2_O_3,_ x = 0.1, 0.5, 1.0, 1.5, 2.0, 2.5	2.64–2.82	[65]
60ZnO:20B_2_O_3_:(20 − x)SiO_2_ − xEu_2_O_3_ (x = 0 and 1)	3.166	[66]
Eu^3+^:Y_2_O_3_	3.8–5.2	[67,68]

**Table 7:** CIE chromaticity coordinates of the borosilicate glasses.

Glass Composition	Chromaticity Coordinates (x, y)	CCT (K)
35B_2_O_3_:35SiO_2_:25La_2_O_3_:5CaO:0.5Eu_2_O_3_	0.646, 0.349	2247.28
32.5B_2_O_3_:32.5SiO_2_:25La_2_O_3_:5CaO:0.5Eu_2_O_3_:5WO_3_	0.651, 0.348	2325.99
30B_2_O_3_:30SiO_2_:25La_2_O_3_:5CaO:0.5Eu_2_O_3_:10WO_3_	0.652, 0.347	2363.19
27.5B_2_O_3_:27.5SiO_2_:25La_2_O_3_:5CaO:0.5Eu_2_O_3_:15WO_3_	0.652, 0.347	2363.19
NTSC standard for red light	0.670, 0.330	
Y_2_O_2_S:Eu^3+^	0.658, 0.340	

## Data Availability

Data are contained within the article. Further inquiries can be directed to the corresponding author.
